# Detection of Intestinal Inflammation by Vascular Adhesion Protein-1-Targeted [^68^Ga]Ga-DOTA-Siglec-9 Positron Emission Tomography in Murine Models of Inflammatory Bowel Disease

**DOI:** 10.1007/s11307-023-01885-8

**Published:** 2023-12-18

**Authors:** Achol A. Bhowmik, Taina R. H. Heikkilä, Lauri Polari, Jenni Virta, Heidi Liljenbäck, Olli Moisio, Xiang-Guo Li, Riikka Viitanen, Sirpa Jalkanen, Jukka Koffert, Diana M. Toivola, Anne Roivainen

**Affiliations:** 1 Turku PET Centre, University of Turku, Kiinamyllynkatu 4-8, FI-20520 Turku, Finland; 2 Cell Biology, Biosciences, Faculty of Science and Engineering, Åbo Akademi University, Turku, Finland; 3 InFLAMES Research Flagship, Åbo Akademi University, Turku, Finland; 4 Turku Center for Disease Modelling, University of Turku, Turku, Finland; 5 Department of Chemistry, University of Turku, Turku, Finland; 6 InFLAMES Research Flagship, University of Turku, Turku, Finland; 7 MediCity Research Laboratory, University of Turku, Turku, Finland; 8 Department of Gastroenterology, Turku University Hospital, Turku, Finland; 9 Turku PET Centre, Turku University Hospital, Turku, Finland

**Keywords:** Inflammatory bowel disease, Murine colitis, Positron emission tomography, Vascular adhesion protein-1, Siglec-9, [^68^Ga]Ga-DOTA-Siglec-9

## Abstract

**Purpose:**

Inflammatory bowel disease (IBD) can be imaged with positron emission tomography (PET), but existing PET radiopharmaceuticals have limited diagnostic accuracy. Vascular adhesion protein-1 (VAP-1) is an endothelial cell surface molecule that controls leukocyte extravasation into sites of inflammation. However, the role of inflammation-induced VAP-1 expression in IBD is still unclear. Therefore, this study investigated the utility of VAP-1-targeted [^68^Ga]Ga-DOTA-Siglec-9 positron emission tomography/computed tomography (PET/CT) for assessing inflammation in two mouse models of IBD.

**Procedures:**

Studies were performed using K8^−/−^ mice that develop a chronic colitis-phenotype and C57Bl/6NCrl mice with acute intestinal inflammation chemically-induced using 2.5% dextran sodium sulfate (DSS) in drinking water. In both diseased and control mice, uptake of the VAP-1-targeting peptide [^68^Ga]Ga-DOTA-Siglec-9 was assessed in intestinal regions of interest using *in vivo* PET/CT, after which *ex vivo* gamma counting, digital autoradiography, and histopathological analyses were performed. Immunofluorescence staining was performed to determine VAP-1-expression in the intestine, including in samples from patients with ulcerative colitis.

**Results:**

Intestinal inflammation could be visualized by [^68^Ga]Ga-DOTA-Siglec-9 PET/CT in two murine models of IBD. In both models, the *in vivo* PET/CT and *ex vivo* studies of [^68^Ga]Ga-DOTA-Siglec-9 uptake were significantly higher than in control mice. The *in vivo* uptake was increased on average 1.4-fold in the DSS model and 2.0-fold in the K8^−/−^ model. Immunofluorescence staining revealed strong expression of VAP-1 in the inflamed intestines of both mice and patients.

**Conclusions:**

This study suggests that the VAP-1-targeting [^68^Ga]Ga-DOTA-Siglec-9 PET tracer is a promising tool for non-invasive imaging of intestinal inflammation. Future studies in patients with IBD and evaluation of the potential value of [^68^Ga]Ga-DOTA-Siglec-9 in diagnosis and monitoring of the disease are warranted.

**Supplementary Information:**

The online version contains supplementary material available at 10.1007/s11307-023-01885-8.

## Introduction

Inflammatory bowel disease (IBD) covers a group of diseases characterized by idiopathic chronic inflammation of the alimentary tract. The two most common subtypes of IBD are Crohn’s disease and ulcerative colitis (UC). Although the exact etiology is not well known, it is generally accepted that IBD develops due to dysregulated immune responses generated by complex interactions of predisposing environmental factors, gut microbiota, and genetic factors [1, 2]. The most common symptoms of IBD are bloody feces, diarrhea, and abdominal pain. Despite the use of non-invasive tests such as fecal calprotectin, endoscopy and tissue biopsies supported by the patient’s medical history, laboratory, and radiological imaging play a fundamental role in the diagnosis of IBD [3, 4]. However, non-definitive diagnostic criteria, the variable appearance of inflammation in the relapsing and remitting course of the disease, and poor compliance with invasive procedures, may limit the diagnostic value of endoscopy [3]. Furthermore, the utility of fecal calprotectin may vary according to the anatomical location of IBD [5]. Therefore, more accurate non-invasive diagnostic tools for the detection, monitoring and quantification of intestinal inflammation are needed.

The fluorine-18-labeled glucose analogue 2-deoxy-2[^18^F]fluoro-*D*-glucose ([^18^F]FDG) is routinely used for non-invasive positron emission tomography (PET) imaging of inflammation. Although [^18^F]FDG PET has shown high sensitivity for the detection of intestinal inflammation [6], physiological intestinal glucose uptake limits its diagnostic accuracy [7]. Therefore, other promising tracers for the detection of intestinal inflammation on PET have recently been studied, such as [^89^Zr]Zr-DFO-infliximab (an anti-tumor necrosis factor alpha antibody) [8, 9].

Vascular adhesion protein-1 (VAP-1) is an endothelial surface molecule that controls leukocyte extravasation from blood vessels into sites of inflammation [10, 11]. Expression of VAP-1 has been described in patients with rheumatoid arthritis and patients with primary sclerosing cholangitis [12, 13], and several publications describe VAP-1-targeted *in vivo* imaging in various experimental disease models [14–16]. However, the role of VAP-1 in PET imaging of intestinal inflammation has not been studied. We previously showed that sialic acid-binding immunoglobulin-like lectin 9 (Siglec-9) is a VAP-1 ligand, and that a gallium-labelled Siglec-9-motif-containing peptide ([^68^Ga]Ga-DOTA-Siglec-9) can be used for PET imaging of inflammation and certain cancers [17]. In this study, we investigated the utility of VAP-1-targeted [^68^Ga]Ga-DOTA-Siglec-9 PET imaging for the detection of intestinal inflammation in both chronic genetic and acute chemically-induced mouse models of IBD. Intestinal uptake of [^68^Ga]Ga-DOTA-Siglec-9 was evaluated using small-animal PET/CT, *ex vivo* studies, and histopathological analysis of colitis. Furthermore, expression of VAP-1 was evaluated by immunofluorescence staining in both mouse and human tissue samples.

## Materials and Methods

### Mouse Models

To induce acute experimental colitis, 2.5% dextran sodium sulfate (DSS; 40 kDa; TdB Labs AB, Uppsala, Sweden) was administered in autoclaved drinking water to 2–4-month-old C57Bl/6NCrl male mice (Charles River Laboratories Inc., Wilmington, MA, USA) for 7 days followed by 1–2 days of DSS-free water [18, 19]. PET/CT scans were obtained on day 8 ([^18^F]FDG) and day 9 ([^68^Ga]Ga-DOTA-Siglec-9) after initiation of DSS dosing. Age- and sex-matched control C57Bl/6NCrl mice received autoclaved drinking water without DSS. The severity of the disease was determined according to a disease activity index (DAI) calculated according to daily measurements (starting on day 0) of body weight loss (1 point for each 5% of body weight loss since the start of the experiment), presence of occult blood in stool (0 = none; 1 = small amounts of blood in stool pellets; 2 = blood found throughout pellet; 3 = clotted blood at anus; 4 = fresh blood on mice or on bedding materials of cage), and stool consistency (1 = normal; 2 = formed but very soft; 3 = slightly loose; 4 = liquid) [19].

For the chronic genetic colitis model, age- and sex-matched female 3–6-month-old keratin 8 (K8) wild-type (K8^+/+^) and K8-knockout (K8^−/−^) female mice with an FVB/n background were used [20]. Absence of K8-protein causes a chronic colitis-phenotype. Mice were genotyped as previously described [21]. Briefly, genotyping was performed by polymerase chain reaction (PCR) using DNA extracted from earpieces. The PCR was followed by agarose gel electrophoresis.

All animal experiments were approved by the national Project Authorization Board in Finland (licenses ESAVI/16359/2019 and ESAVI/8648/2020) and were carried out in compliance with EU Directive 2010/EU/63 on the protection of animals used for scientific purposes. Mice were housed at the Central Animal Laboratory of University of Turku under standard conditions (12 h light/dark cycle) with ad libitum access to a standard diet and water*.* A study flow chart is shown in Supplementary Fig. 1.

### Radiochemistry

[^68^Ga]Ga-DOTA-Siglec-9 was prepared according to a previously described procedure [16]. The DOTA-Siglec-9 precursor compound (Peptide Specialty Laboratories GmbH, Heidelberg, Germany) is a cyclic peptide consisting of 17 amino acid residues in the sequence CARLSLSWRGLTLCPSK, with a disulfide bond between cysteine residues 1 and 14. For ^68^Ga-labeling, the DOTA chelator was attached to the C-terminus and DOTA denotes 1,4,7,10-tetraazacyclododecane-′*N*,*N*′,*N*″,*N*‴-tetraacetic acid. In addition, an 8-amino-3,6-diooxaoctanoyl linker (polyethylene glycol derivative) was inserted between DOTA and the peptide sequence. The radiochemical purity was more than 95% in all batches throughout the study, as analyzed by high-performance liquid chromatography, and molar activity was 22.0 ± 5.8 GBq/µmol.

### *In Vivo *PET/CT Imaging

Small-animal PET and CT systems (Molecubes NV, Gent, Belgium) were used for *in vivo* imaging, with the mice anesthetized with isoflurane. The tail vein was cannulated and the urinary bladder of the female mice was catheterized before imaging. The mice were injected with [^68^Ga]Ga-DOTA-Siglec-9 (10.1 ± 2.7 MBq, 2.2 ± 1.4 µg, 0.9 ± 0.6 nmol) via the tail vein and a 60-min dynamic PET acquisition (six 10-s, four 60-s. five 300-s, and three 600-s time frames) was acquired. In addition, for anatomical reference and attenuation correction, 100 µL of iodinated contrast agent (eXia 160XL; Binitio Biomedical Inc., Ottawa, ON, Canada) was injected intravenously (i.v.) immediately after the [^68^Ga]Ga-DOTA-Siglec-9 PET and high-resolution CT imaging was acquired.

The intestinal tract was visualized as a metabolic reference, with a 20-min static [^18^F]FDG (3.5 ± 0.6 MBq) PET acquisition starting 40 min after injection being acquired the day before the [^68^Ga]Ga-DOTA-Siglec-9 study.

Quantitative PET analysis was performed using Carimas software (version 2.10; Turku PET Centre, Turku, Finland). Region of interest (ROI) was defined in the distal colon on [^68^Ga]Ga-DOTA-Siglec-9 PET images co-registered with CT (Supplementary Fig. 2) and supported by [^18^F]FDG PET images. The uptake of [^68^Ga]Ga-DOTA-Siglec-9 was reported as the mean standardized uptake value (SUV_mean_), which was calculated as the average radioactivity concentration of the ROI corrected for the injected radioactivity dose and animal weight.

The association between colonic *in vivo* [^68^Ga]Ga-DOTA-Siglec-9 PET uptake and the histopathological inflammation score was determined by correlation analyses. Furthermore, time-activity values were determined to the distal colon.

### *Ex Vivo* Biodistribution and Autoradiography

Immediately after the [^68^Ga]Ga-DOTA-Siglec-9 PET/CT imaging, blood was drawn by cardiac puncture and mice were euthanized by cervical dislocation. The intestinal tract was excised, blood and contents carefully removed, and weighed. Other organs were also collected and weighed for *ex vivo* biodistribution studies. The radioactivity concentration of the tissues was measured using a gamma counter (Triathler 3″; Hidex, Turku, Finland). The measurements were corrected for radionuclide decay to the time of injection and the weight of the tissue, and the results were expressed as percentage of injected radioactivity dose per gram of tissue (%ID/g).

To evaluate uptake of [^68^Ga]Ga-DOTA-Siglec-9 in the intestinal tract with more precision, 20-µm cryosections were examined using digital autoradiography. After gamma counting, excised segments of ileum and proximal colon and three segments of distal colon were embedded in TissueTek (Sakura Finetek, Alphen aan den Rijn, The Netherlands), frozen in dry ice-cooled isopentane, and cut into sequential transverse 5-µm, 8-µm, and 20-µm slices. The 20-µm sections were then thaw-mounted onto microscope slides and apposed on an imaging plate (Fuji Imaging Plate BAS-TR2025; Fujifilm Corp., Tokyo, Japan). The imaging plate was scanned after an exposure time of 2.5 h (Fuji Analyzer BAS-5000; Fujifilm Corp., Tokyo, Japan; internal resolution 25-µm). After scanning, sections were stored at − 70 °C until hematoxylin–eosin (H&E) staining.

After superimposing autoradiographs and H&E stained images, tracer uptake was determined in ROIs of bowel wall segments containing mucosa and submucosa from diseased and control mice. The results were expressed as average photostimulated luminescence per square millimeter (PSL/mm^2^) using Carimas software. The accumulation for background radiation was subtracted from the ROI data and the results were decay-corrected for injection time and exposure time and normalized for injected radioactivity dose.

### Histopathology and Immunofluorescence Staining

For histopathological analyses, 5-µm and 20-µm cryosections were stained with H&E, scanned with a digital slide scanner (Pannoramic Midi; 3DHistec Ltd., Budapest, Hungary), and analyzed with CaseViewer software (version 2.2; 3DHistec Ltd., Budapest, Hungary). The severity of colonic inflammation was scored as: 0 = normal tissue, 1 = minor increase in the number of inflammatory cells in mucosa, 2 = moderate infiltration, and 3 = severe inflammation with topical neutrophils. Hyperproliferation was scored as 0–3 according to crypt elongation, with 0 = normal crypt length and 3 = longest crypts. Edema was scored according to the number and size of edematous regions, ranging from 0 = no edema, to 3 = extended edema. Tissue erosion depth was scored as: 0 = no erosion, 1 = topical erosion, 2 = erosion of the mucous layer, and 3 = erosion through the muscular lamina [22]. Two people (authors AAB and LP, the latter of whom being an experienced murine gastrointestinal pathology researcher) analyzed histological changes independently and the presented scores represent averages of these analyses. The histopathological score was formed as the sum of the scores for inflammation, hyperproliferation, edema, and erosion.

To detect luminal VAP-1 expression, mice were i.v. injected with a monoclonal rat anti-mouse VAP-1 antibody (7–88, 1 mg/kg diluted in saline) 10 min before being euthanized [23]. Cryosections of 8 µm were stained with secondary anti-rat immunoglobulin conjugated to a fluorescent dye (Alexa Fluor 488; Invitrogen, Waltham, MA, USA) and scanned with a digital slide scanner. VAP-1 expression in bowel wall segments from diseased and control tissues was visually assessed with CaseViewer software.

### Human Tissue Samples

Frozen sections from intestinal biopsies of patients with UC who were enrolled in a clinical trial from 2000–2001 (CLN9801, ethics approval 31.3.1998 §76, and amendment CLN9901 16.3.1999 §97) were cut into 6-µm sections and stained with fluorescein isothiocyanate-conjugated anti-mouse IgM antibody (Southern Biotechnology Associates Inc., Birmingham, AL, USA). The patients (*n* = 3 in each dose cohort) received an intravenous infusion of 0.05, 0.2, or 0.5 mg/kg of monoclonal mouse anti-human VAP-1 antibody (Vepalimomab, IgM) before the colonoscopy and biopsies. A comparable protocol regarding the manufacturing and dosing of the antibody was used in patients with contact dermatitis [24].

### Statistical Analyses

All values are presented as mean ± standard deviation (SD). Independent samples *t* tests were used to compare two groups. Two-way ANOVA and Bonferroni post hoc tests were used for repeated measurements of the DSS study follow-up data. Nonparametric Spearman’s and parametric Pearson’s correlation coefficients were used for correlation analyses. *P* values less than 0.05 were considered statistically significant. Statistical analyses were performed with IBM SPSS Statistics (version 28; IBM Corp., Armonk, NY, USA) and GraphPad Prism (version 10.0.2 for Windows; GraphPad Software, Boston, MA, USA).

## Results

### Effects of DSS-induced Colitis

**Fig. 1:**
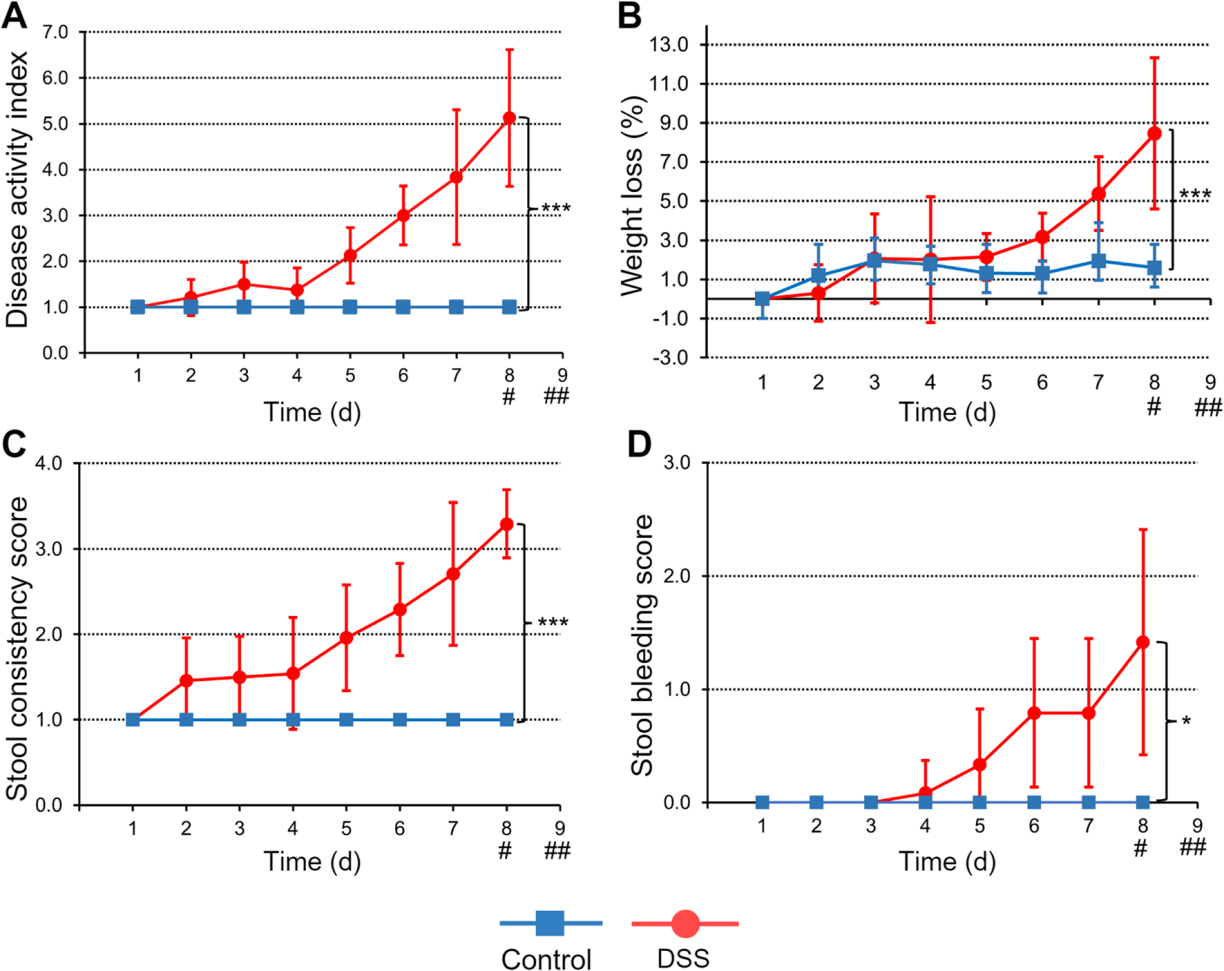
The course of the DSS-induced colitis for male C57Bl/6Ncrl mice that received 2.5% DSS in drinking water for the first 7 days (H_2_O for controls) then 1–2 days with H_2_O. Controls are healthy C57Bl/6Ncrl mice without DSS induction. (**A**) The disease activity index value is combination of weight loss, stool consistency, and presence of occult blood in stool. (**B**) Body weight loss is expressed as a percentage of weight at the start of the experiment. Individual graphs for (**C**) stool consistency and (**D**) occult stool blood are shown. Values are expressed as mean ± SD. Statistical significance was calculated with two-way ANOVA and Bonferroni post hoc test. *P < 0.05. ***P < 0.001. ^#^[^18^F]FDG-PET/CT. ^##^[^68^Ga]Ga-DOTA-Siglec-9-PET/CT.

The colitis DAI was significantly higher in DSS mice than in healthy controls (5.1 ± 1.5 vs. 1.0 ± 0.0 [day 8], *P* < 0.001; Fig. 1A). DSS mice developed weight loss (Fig. 1B), diarrhea (Fig. 1C), and occult blood in feces (Fig. 1D). DSS mice showed greater body weight loss than healthy controls at the beginning of the *in vivo* imaging that was performed on day 8 (8.4% ± 3.9% vs. 1.6% ± 1.2% [day 8], *P* < 0.001; Fig. 1B).

### Histopathology of Intestinal Inflammation

**Fig. 2:**
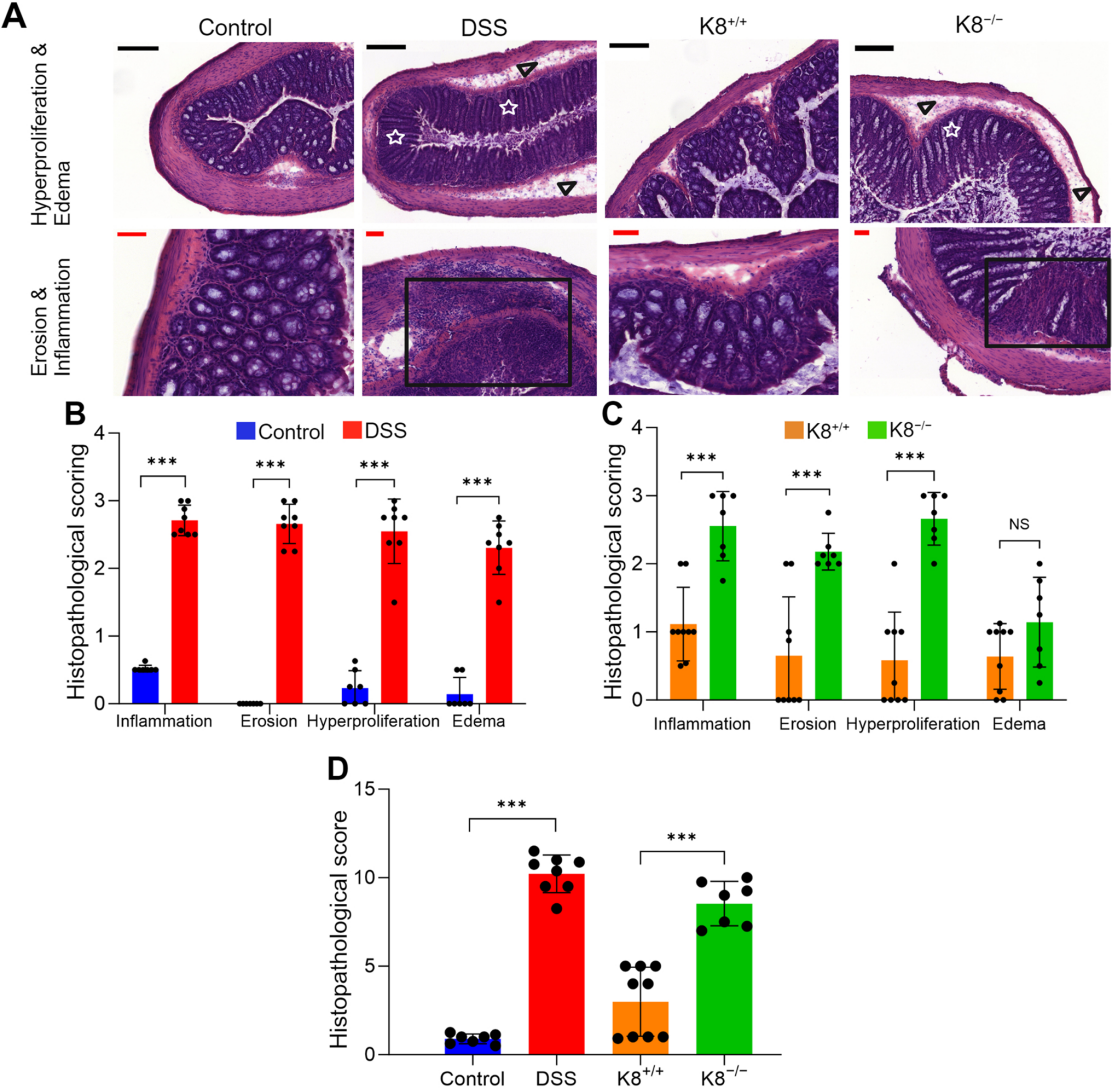
Histopathological scoring showed increased inflammation in the distal colon of two colitis models. The histopathological features in the distal colon were scored from hematoxylin–eosin (H&E) stained tissue sections (representative images and features are shown in **A**). The presence of erosion (**A**, black boxes), inflammatory cells, hyperproliferation (**A**, white stars), and edema (**A**, black arrowheads) were scored on a scale from zero to three in both (**B**) the DSS-induced colitis model and (**C**) K8^−/−^ model. (**D**) The histopathological score is the sum of the scoring criteria. Values are presented as mean ± SD. ***P < 0.001, independent samples *t* test. NS, not significant. The black scale bars are 200 µm and the red scale bars are 50 µm.

Colon sections from the DSS and K8^−/−^ colitis models and controls were H&E stained (Fig. 2A) and subjected to histopathological scoring (Fig. 2B − D). Histopathological analyses showed increased inflammation in the distal colon of DSS mice and K8^−/−^ mice compared with their respective controls (inflammation score 2.9 ± 0.2 vs. 0.5 ± 0.0 in DSS model, *P* < 0.001; Fig. 2B; inflammation score 2.6 ± 0.5 vs. 1.1 ± 0.5 in K8^−/−^ model, *P* < 0.001; Fig. 2C). The histopathological score was higher on average in DSS mice (10.6 ± 0.9; Fig. 2D) than in K8^−/−^ mice (8.5 ± 1.3; Fig. 2D). The extension of mucosal defect was more limited to mucosa in K8^−/−^ mice (erosion score 2.2 ± 0.3 vs. 0.7 ± 0.9, *P* < 0.001; Fig. 2C), whereas in most DSS mice, the muscular lamina was damaged (erosion score 2.8 ± 0.2 vs. 0.0 ± 0.0, *P* < 0.001; Fig. 2C).

### *In Vivo *Uptake of [^68^Ga]Ga-DOTA-Siglec-9 in Intestinal Inflammation

**Fig. 3:**
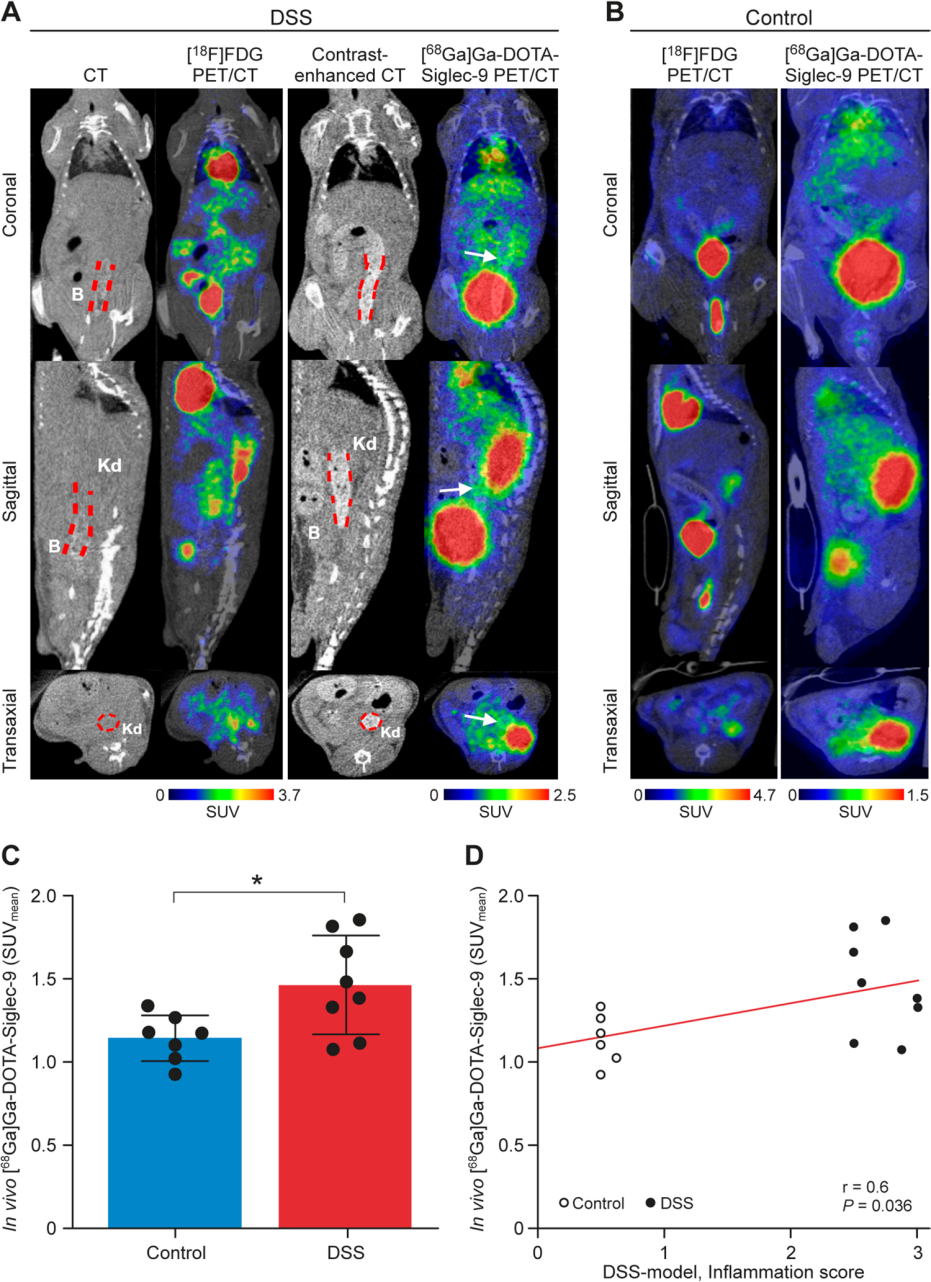
*In vivo* imaging of DSS and control male mice. (**A**) Representative CT images, contrast-enhanced CT images, fused [^18^F]FDG PET/CT images (time-weighted mean of PET frames from 40-60 min post-injection), and fused [^68^Ga]Ga-DOTA-Siglec-9 PET/CT images (time-weighted mean of PET frames from 0-60 min post-injection) of the same DSS mouse. *In vivo* [^68^Ga]Ga-DOTA-Siglec-9 binding in the distal colon is enhanced (white arrows). (**B**) Representative *in vivo* PET/CT images of non-DSS healthy control male mouse (C57Bl/6NCrl). (**C**) Quantification of *in vivo* [^68^Ga]Ga-DOTA-Siglec-9 PET signal is presented as mean ± SD. *P* value is from independent samples *t* test. *P < 0.05. (**D**) Correlation analysis between [^68^Ga]Ga-DOTA-Siglec-9 uptake in distal colon and histopathological inflammation score. Pearson’s correlation coefficient (*r*) and *P* value are presented. SUV, standardized uptake value. Red dashed lines, distal colon; B, urinary bladder; Kd, kidney.

**Fig. 4:**
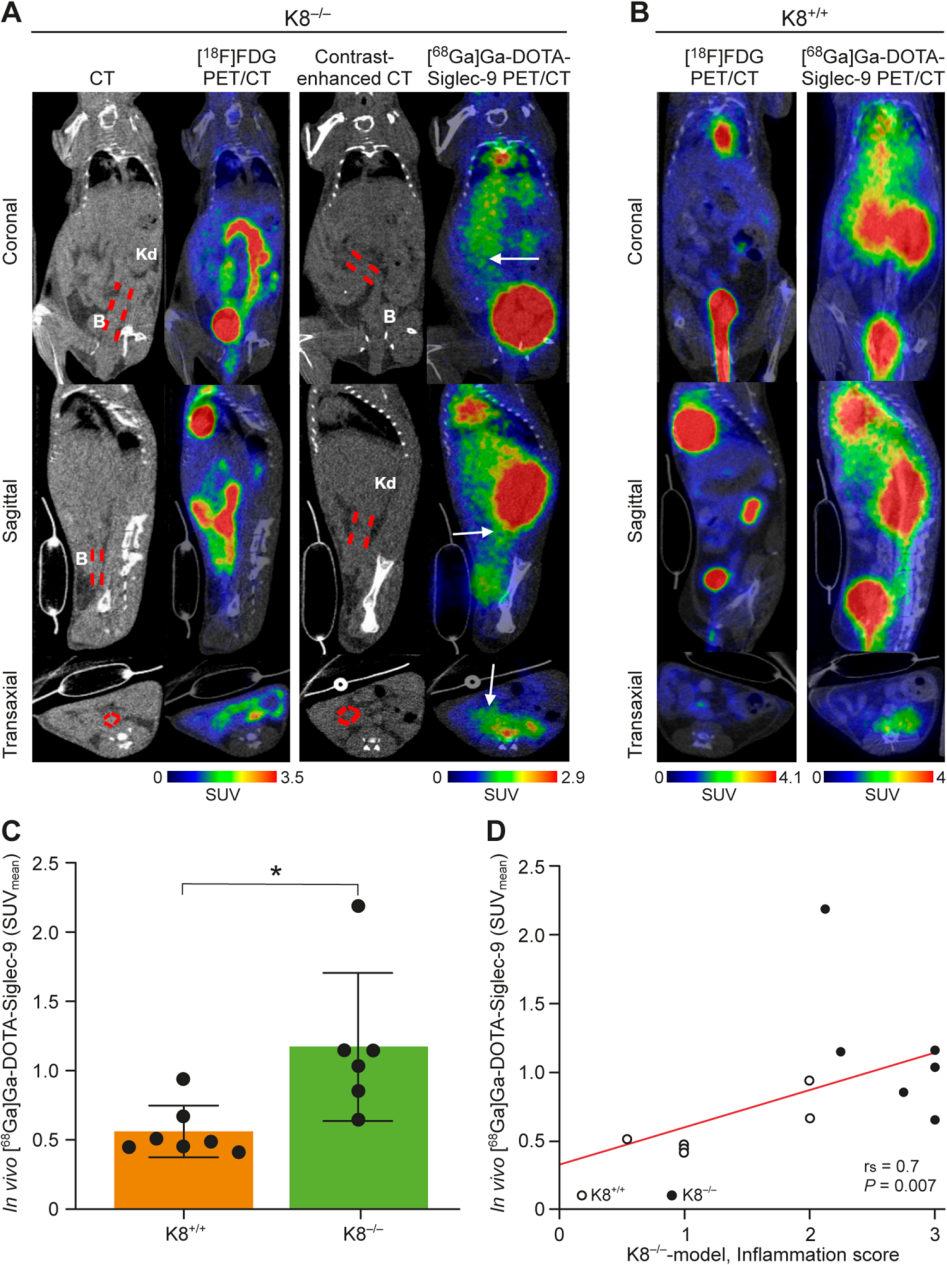
*In vivo* imaging of K8^−/−^ and control female mice. (**A**) Representative CT images, contrast-enhanced CT images, fused [^18^F]FDG PET/CT images (time-weighted mean of PET frames from 40-60 min post-injection), and fused [^68^Ga]Ga-DOTA-Siglec-9 PET/CT images (time-weighted mean of PET frames from 0-60 min post-injection) of the same mouse. *In vivo* [^68^Ga]Ga-DOTA-Siglec-9 binding in the distal colon is enhanced (white arrows). (**B**) Representative *in vivo* PET/CT images of healthy K8^+/+^ female mouse (FVB/n). (**C**) Quantification of *in vivo* [^68^Ga]Ga-DOTA-Siglec-9 PET signal is presented as mean ± SD. *P* values is from independent samples *t* tests. *P < 0.05. (**D**) Correlation analyses between [^68^Ga]Ga-DOTA-Siglec-9 uptake in distal colon and histopathological inflammation score. Spearman’s correlation coefficient (*r*_*s*_) and *P* value are presented. SUV, standardized uptake value. Red dashed lines, distal colon; B, urinary bladder; Kd, kidney.

Histologically-proven intestinal inflammation in the distal colon of the DSS (Fig. 3A) and K8^−/−^ mice (Fig. 4A) was visible with [^68^Ga]Ga-DOTA-Siglec-9 PET/CT, whereas their respective controls showed only low tracer uptake. [^18^F]FDG PET/CT used as a reference visualized metabolically active parts of small intestine and colon (Fig. 3A-B and Fig. 4A-B). [^68^Ga]Ga-DOTA-Siglec-9 uptake in the distal colon was significantly higher in both chemically-induced and genetic colitis mice than in control mice (SUV_mean_, 1.5 ± 0.3 vs. 1.1 ± 0.1 in DSS model, *P* = 0.021; Fig. 3C; and SUV_mean_, 1.2 ± 0.5 vs. 0.6 ± 0.2 in K8^−/−^ model, *P* = 0.024; Fig. 4C). The individual [^68^Ga]Ga-DOTA-Siglec-9 uptake in the distal colon correlated significantly with the histopathological inflammation score (*r* = 0.6 in DSS model, *P* = 0.036; Fig. 3D and *r*_*s*_ = 0.7 in K8^−/−^ model, *P* = 0.007; Fig. 4D). Decay-corrected time-activity curves of the distal colon are shown in Supplementary Fig. 3.

### *Ex Vivo* Biodistribution and Autoradiography

**Fig. 5:**
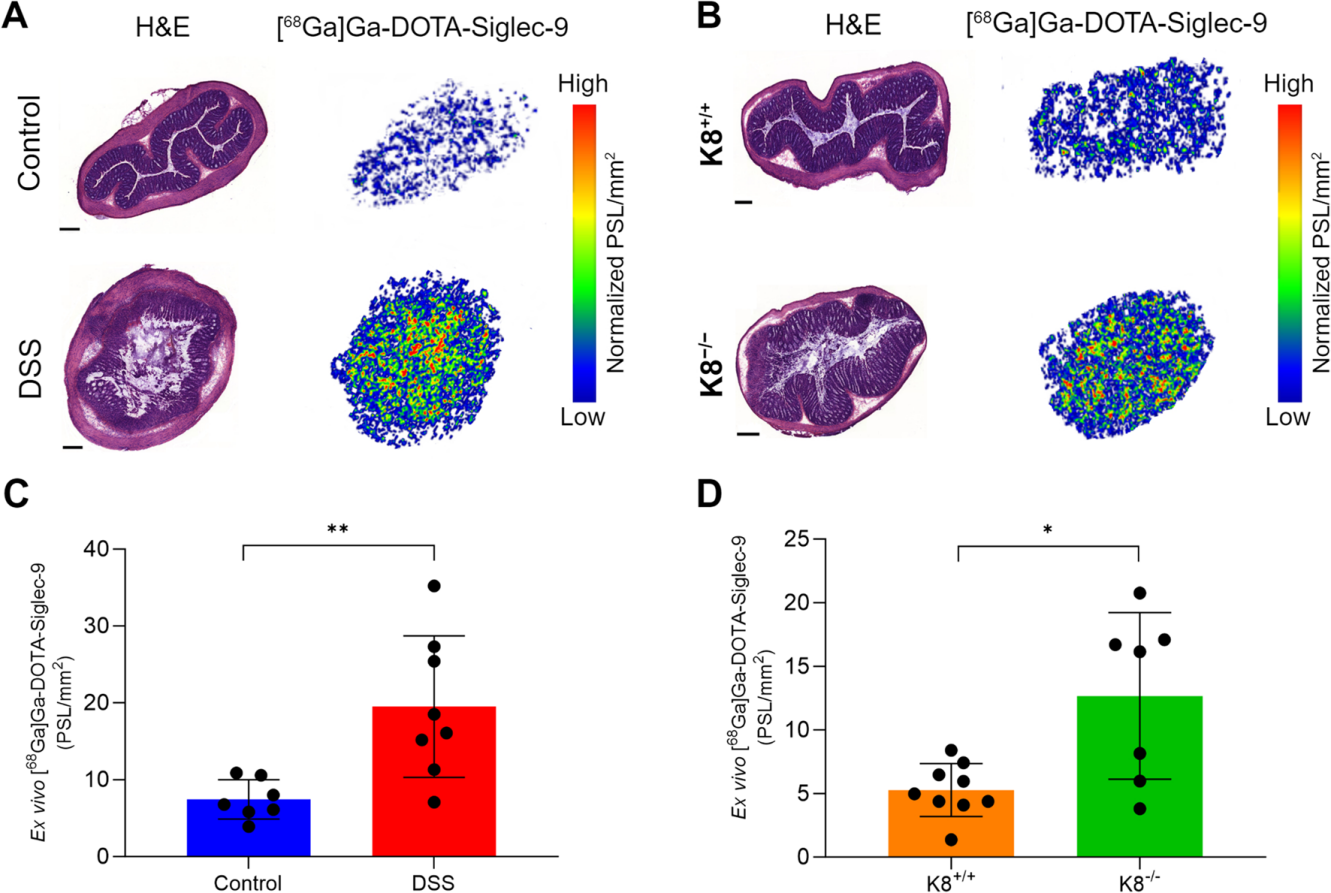
Quantification of [^68^Ga]Ga-DOTA-Siglec-9 binding by *ex vivo* autoradiography of colon sections from two colitis models. Representative cross-sectional hematoxylin–eosin (H&E) staining and *ex vivo* autoradiographs of 20-µm cryosections of distal colon bowel wall mucosa and submucosa in (**A**) the DSS-induced colitis model and (**B**) K8^−/−^ model. *Ex vivo* autoradiography of colon sections shows increased tracer uptake in colonic mucosa and submucosa from two colitis models (**C** and **D**) compared to healthy control C57Bl/6NCrl and K8^+/+^ mice. Values are presented as mean ± SD. *P* values are from independent samples *t* tests. *P < 0.05, **P < 0.01. PSL/mm^2^, photostimulated luminescence per square millimeter normalized for injected radioactivity dose. The scale bars are 250 µm.

The *ex vivo* digital autoradiography analysis of intestinal inflammation confirmed the *in vivo* [^68^Ga]Ga-DOTA-Siglec-9 PET/CT imaging data. Autoradiography revealed high uptake of [^68^Ga]Ga-DOTA-Siglec-9 in both DSS mice and K8^−/−^ mice, whereas only low uptake was detected in their respective controls (19.8 ± 9.5 vs. 7.4 ± 2.6 PSL/mm^2^ in DSS model, *P* = 0.005; 12.7 ± 6.6 vs. 5.3 ± 2.1 PSL/mm^2^ in K8^−/−^ model, *P* = 0.024; Fig. 5). The *ex vivo* biodistribution results of [^68^Ga]Ga-DOTA-Siglec-9 are presented in Table 1.

**Table 1:** *Ex vivo* biodistribution of [^68^Ga]Ga-DOTA-Siglec-9 in mice at 60 min post-injection

	Control (*n* = 7)	DSS (*n* = 8)	*P* value	K8^+/+^ (*n* = 9)	K8^−/−^ (*n* = 7)	*P* value
Distal colon	0.32 ± 0.22	1.03 ± 0.71	0.026*	0.27 ± 0.10	0.52 ± 0.23	0.009 **
Proximal colon	0.28 ± 0.10	0.51 ± 0.30	0.076	0.25 ± 0.11	0.40 ± 0.17	0.048*
Small intestine	0.24 ± 0.14	0.43 ± 0.29	0.142	0.22 ± 0.10	0.36 ± 0.13	0.020*

Results are expressed as percentage of injected radioactivity dose per gram of tissue, mean ± SD. *P* values are from independent samples* t* tests. *P < 0.05, **P < 0.01

### VAP-1 Expression in Murine Colitis and Human Patients with IBD

**Fig. 6:**
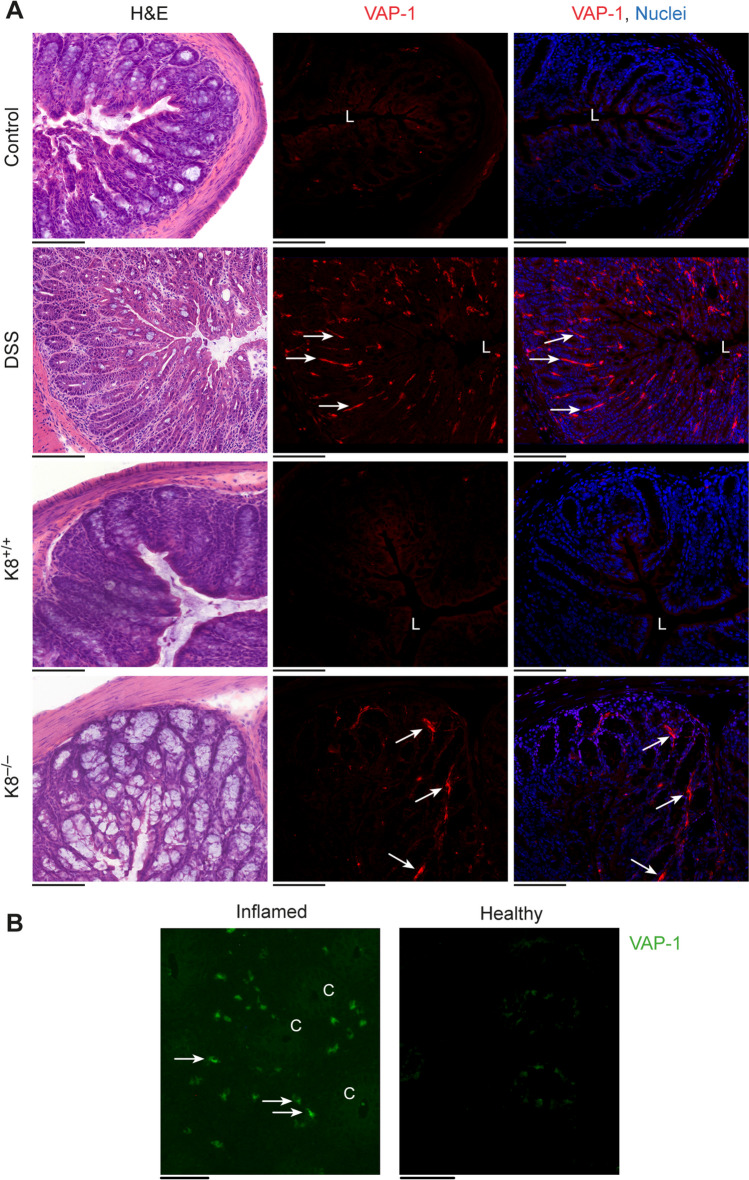
Vascular adhesion protein-1 (VAP-1) is expressed in intestinal inflammation. (**A**) Representative VAP-1 immunofluorescence and H&E staining of colon samples from diseased mice. All the mice were i.v. injected with a monoclonal rat anti-mouse VAP-1 antibody (7–88) 10 min before being sacrificed. (**B**) Representative VAP-1 immunofluorescence staining of inflamed and healthy distal colon samples from a patient with ulcerative colitis who received anti-VAP-1 antibody (0.5 mg/kg) before biopsy. C, crypt; L, lumen. White arrows denote VAP-1-positivity. The scale bars are 100 µm.

Tissue samples of mice with histologically confirmed colitis showed VAP-1 expression in the inflammatory lesions. Using immunofluorescence staining, high VAP-1 expression was detected in the venules of the lamina propria of inflamed distal colon (Fig. 6A), whereas only low expression was detected in the submucosal venules of healthy control mice.

We next analyzed whether VAP-1 is translocated to the endothelial cell surface in patients with UC. When the VAP-1 antibody was administered intravenously and only the secondary antibody was applied for immunofluorescence staining, our results revealed VAP-1 on the endothelial surface in inflamed areas of the colon (Fig. 6B).

## Discussion

Numerous studies have shown that VAP-1 is rapidly translocated to the endothelial cell surface upon inflammation, where it mediates leukocyte trafficking from the blood into the inflamed tissue [10, 11]. Our justification for this current study was to explore a novel approach to detect intestinal inflammation in mouse models of IBD. In this study, we used both *in vivo* PET/CT and *ex vivo* autoradiography and gamma counting to demonstrate that VAP-1-targeted [^68^Ga]Ga-DOTA-Siglec-9 can detect intestinal inflammation in two mouse models, with this inflammation being most pronounced in the distal part of the colon. Furthermore, colonic [^68^Ga]Ga-DOTA-Siglec-9 uptake correlated significantly with histologically confirmed inflammation score. Our results suggest that VAP-1-targeting [^68^Ga]Ga-DOTA-Siglec-9 PET/CT is feasible for the evaluation of murine colitis, and therefore has potential as a non-invasive diagnostic tool for imaging patients with IBD.

The DSS-induced colitis model and K8^−/−^ mouse model are well-established experimental colitis models used for studies investigating intestinal inflammation [21, 25–27]. The DSS model severity is dose, sex and age dependent. Here, male DSS mice were used, based on experience with the model severity and progression in previous research at our institute [22]. Female K8^−/−^ mice were used in the study based on their availability. No major differences in the colitis phenotype between male and female in this model have been seen or reported [21]. The mouse models mimic some of the typical IBD symptoms that were used to observe inflammatory activity, such as weight loss and rectal bleeding. Although these clinical parameters can be repeatedly and noninvasively measured, they do not necessarily show the severity of the inflammatory process in the intestinal tract.

Several studies have demonstrated PET imaging using the glucose analogue [^18^F]FDG for detecting intestinal inflammation in murine models [28–30], and in accord with these studies, we were able to detect high uptake of [^18^F]FDG in the alimentary tract of diseased mice. However, [^18^F]FDG may give false-positive results due to variable physiological uptake in the intestines (e.g., lumen and microbiota), which limits direct comparison with [^68^Ga]Ga-DOTA-Siglec-9. Generator-produced ^68^Ga enables radiolabeling via various linkers to create many peptide-based PET tracers in a more straightforward and cost-effective way than radiolabeling with cyclotron-produced radionuclides [31]. ^68^Ga-labelled tracers may offer potential replacements for [^18^F]FDG in the imaging of intestinal inflammation. However, investigations of inflammation-selective radiopharmaceuticals need to be extended to clinical studies.

There are some limitations to our study. Rapid excretion of [^68^Ga]Ga-DOTA-Siglec-9 through kidneys to the urinary bladder may have partially limited the *in vivo* analysis by creating a signal spillover effect in the distal colon. However, signal spillover in the urinary bladder of human could be reduced with frequent bladder voids or bladder emptying with urinary catheter. The highest uptake of [^68^Ga]Ga-DOTA-Siglec-9 was seen in the kidneys and urinary bladder, consistent with elimination via the urinary system [13–17].

Previously, Salmi and co-workers reported that VAP-1 is normally weakly expressed in the blood vessels of the intestinal mucosa, but is rapidly induced in the inflammatory lesions of IBD [11]. MAdCAM-1 is an endothelial cell adhesion molecule that recruits leukocytes from blood vessels to inflamed and damaged mucosa in the intestinal tract of IBD patients [32]. Data from Liaskou and co-workers demonstrated that semicarbazide-sensitive amine oxidase (SSAO) enzyme activity displayed by VAP-1 increases MAdCAM-1 expression in mucosal vasculature *in vivo* [33]. Thus, expression of MAdCAM-1, which increases VAP-1/SSAO activity, leads to uncontrolled recruitment of mucosal effector cells, leading to the tissue damage characteristic of IBD [33]. Other gastrointestinal diseases, such as malignancy and infection, can cause inflammation of the intestines. However, serum and colorectal tissue VAP-1 levels are lower in patients with colorectal cancer [34]. Although our results and previous studies [32, 33] suggest that VAP-1 is a potential PET imaging target for intestinal inflammation in mouse models, the role of VAP-1 in the pathogenesis of IBD and in other inflammatory gastrointestinal diseases remains to be studied.

With noncurative solutions for IBD, treatments are focused on inducing remission, and early diagnosis and monitoring of the disease is essential for effective treatment. The feasibility of [^68^Ga]Ga-DOTA-Siglec-9 PET/CT for assessing the efficacy of therapeutic interventions requires further investigation with reproducible disease models.

## Conclusion

Taken together, our study supports the use of VAP-1 targeting tracer for the imaging of intestinal inflammation, and future studies on [^68^Ga]Ga-DOTA-Siglec-9 as a tool for diagnosis and disease monitoring in patients with IBD are warranted. [^68^Ga]Ga-DOTA-Siglec-9 PET/CT is an efficient non-invasive imaging modality for evaluating experimental murine colitis, and therefore has promising potential in the clinical diagnosis of IBD.

### Supplementary Information

Below is the link to the electronic supplementary material.

Supplementary file1 (PDF 293 KB)

## Data Availability

The analyses of the data supporting the conclusions of this article are included within the article and the supplementary file. The raw datasets used and analyzed during the current study are available from the corresponding author on reasonable request.

## References

[1] Rosen MJ, Dhawan A, Saeed SA (2015). Inflammatory bowel disease in children and adolescents. JAMA Pediatr.

[2] Chang JT (2020). Pathophysiology of inflammatory bowel diseases. Longo DL, ed. N Engl J Med.

[3] Senore C, Bellisario C, Hassan C (2016). Organization of surveillance in GI practice. Best Pract Res Clin Gastroenterol.

[4] Spiceland CM, Lodhia N (2018). Endoscopy in inflammatory bowel disease: Role in diagnosis, management, and treatment. World J Gastroenterol.

[5] Mosli MH, Zou G, Garg SK (2015). C-Reactive protein, fecal calprotectin, and stool lactoferrin for detection of endoscopic activity in symptomatic inflammatory bowel disease patients: A systematic review and meta-analysis. Am J Gastroenterol.

[6] Treglia G, Quartuccio N, Sadeghi R (2013). Diagnostic performance of Fluorine-18-Fluorodeoxyglucose positron emission tomography in patients with chronic inflammatory bowel disease: A systematic review and a meta-analysis. J Crohns Colitis.

[7] Glaudemans AWJM, de Vries EFJ, Galli F (2013). The use of ^18^F-FDG-PET/CT for diagnosis and treatment monitoring of inflammatory and infectious diseases. Clin Dev Immunol.

[8] Seo M, Kim Y, Ye BD (2022). PET imaging of system x_C_^−^ in immune cells for assessment of disease activity in mice and patients with inflammatory bowel disease. J Nucl Med.

[9] Yan G, Wang X, Fan Y (2022). Immuno-PET imaging of TNF-α in colitis using ^89^Zr-DFO-infliximab. Mol Pharm.

[10] Salmi M, Jalkanen S (1992). A 90-kilodalton endothelial cell molecule mediating lymphocyte binding in humans. Science.

[11] Salmi M, Kalimo K, Jalkanen S (1993). Induction and function of vascular adhesion protein-1 at sites of inflammation. J Exp Med.

[12] Trivedi PJ, Tickle J, Vesterhus MN (2018). Vascular adhesion protein-1 is elevated in primary sclerosing cholangitis, is predictive of clinical outcome and facilitates recruitment of gut-tropic lymphocytes to liver in a substrate-dependent manner. Gut.

[13] Viitanen R, Moisio O, Lankinen P (2021). First-in-humans study of ^68^Ga-DOTA-Siglec-9, a PET ligand targeting vascular adhesion protein 1. J Nucl Med.

[14] Virtanen H, Autio A, Siitonen R (2015). ^68^Ga-DOTA-Siglec-9 – a new imaging tool to detect synovitis. Arthritis Res Ther.

[15] Siitonen R, Pietikäinen A, Liljenbäck H (2017). Targeting of vascular adhesion protein-1 by positron emission tomography visualizes sites of inflammation in Borrelia burgdorferi-infected mice. Arthritis Res Ther.

[16] Viitanen R, Virtanen H, Liljenbäck H (2022). [^68^Ga]Ga-DOTA-Siglec-9 detects pharmacodynamic changes of FAP-targeted IL2 variant immunotherapy in B16-FAP melanoma mice. Front Immunol.

[17] Aalto K, Autio A, Kiss EA (2011). Siglec-9 is a novel leukocyte ligand for vascular adhesion protein-1 and can be used in PET imaging of inflammation and cancer. Blood.

[18] Rose WA, Sakamoto K, Leifer CA (2012). Multifunctional role of dextran sulphate sodium for in vivo modeling of intestinal diseases. BMC Immunol.

[19] Breynaert C, Dresselaers T, Perrier C (2013). Unique gene expression and MR T2 relaxometry patterns define chronic murine dextran sodium sulphate colitis as a model for connective tissue changes in human Crohn’s disease. PLoS ONE.

[20] Habtezion A, Toivola DM, Butcher EC (2005). Keratin-8-deficient mice develop chronic spontaneous Th2 colitis amenable to antibiotic treatment. J Cell Sci.

[21] Baribault H, Penner J, Iozzo RV (1994). Colorectal hyperplasia and inflammation in keratin 8-deficient FVB/N mice. Genes Dev.

[22] Polari L, Anttila S, Helenius T (2019). Novel selective estrogen receptor modulator ameliorates murine colitis. Int J Mol Sci.

[23] Merinen M, Irjala H, Salmi M (2005). Vascular adhesion protein-1 is involved in both acute and chronic inflammation in the mouse. Am J Pathol.

[24] Vainio PJ, Kortekangas-Savolainen O, Mikkola JH (2005). Safety of blocking vascular adhesion protein-1 in patients with contact dermatitis. Basic Clin Pharmacol Toxicol.

[25] Eichele DD, Kharbanda KK (2017). Dextran sodium sulphate colitis murine model: An indispensable tool for advancing our understanding of inflammatory bowel diseases pathogenesis. World J Gastroenterol.

[26] Toivola DM, Krishnan S, Binder HJ (2004). Keratins modulate colonocyte electrolyte transport via protein mistargeting. J Cell Biol.

[27] Asghar MN, Priyamvada S, Nyström JH (2016). Keratin 8 knockdown leads to loss of the chloride transporter DRA in the colon. Am J Physiol-Gastrointest Liver Physiol.

[28] Hindryckx P, Staelens S, Devisscher L (2011). Longitudinal quantification of inflammation in the murine dextran sodium sulphate-induced colitis model using μPET/CT. Inflamm Bowel Dis.

[29] Bettenworth D, Reuter S, Hermann S (2013). Translational ^18^F-FDG PET/CT imaging to monitor lesion activity in intestinal inflammation. J Nucl Med.

[30] Asghar MN, Emani R, Alam C (2014). In vivo imaging of reactive oxygen and nitrogen species in murine colitis. Inflamm Bowel Dis.

[31] Vorster M (2023). Gallium-68 labelled radiopharmaceuticals for imaging inflammatory disorders. Semin Nucl Med.

[32] Briskin M, Winsor-Hines D, Shyjan A (1997). Human mucosal addressin cell adhesion molecule-1 is preferentially expressed in intestinal tract and associated lymphoid tissue. Am J Pathol.

[33] Liaskou E, Karikoski M, Reynolds GM (2011). Regulation of mucosal addressin cell adhesion molecule 1 expression in human and mice by vascular adhesion protein 1 amine oxidase activity. Hepatology.

[34] Ward ST, Weston CJ, Shepherd EL (2016). Evaluation of serum and tissue levels of VAP-1 in colorectal cancer. BMC Cancer.

