# Supplementary material for: Detection of Intestinal Inflammation by Vascular Adhesion Protein-1-Targeted [68Ga]Ga-DOTA-Siglec-9 Positron Emission Tomography in Murine Models of Inflammatory Bowel Disease
Source: Mol Imaging Biol. 2023 Dec 18;26(2):322–33. doi: 10.1007/s11307-023-01885-8 (PMC10973022; doi:10.1007/s11307-023-01885-8)
Supplement: Supplementary file 1 — Supplementary file1 (PDF 293 KB) [file 11307_2023_1885_MOESM1_ESM.pdf]

Supplementary Information to

Detection of Intestinal Inflammation by Vascular Adhesion Protein-1-Targeted [^{68}Ga]Ga-DOTA-Siglec-9 Positron Emission Tomography in Murine Models of Inflammatory Bowel Disease

Achol A. Bhowmik[1], Taina R.H. Heikkilä[2,3], Lauri Polari[2,3], Jenni Virta[1], Heidi Liljenbäck[1,4], Olli Moisio[1], Xiang-Guo Li[1,5,6], Riikka Viitanen[1], Sirpa Jalkanen[6,7], Jukka Koffert[1,6,8*], Diana M. Toivola[2,3,4*], Anne Roivainen[1,4,6,9*]

[1]Turku PET Centre, University of Turku, Turku, Finland; [2]Cell Biology, Biosciences, Faculty of Science and Engineering, Åbo Akademi University, Turku, Finland; [3]InFLAMES Research Flagship, Åbo Akademi University, Turku, Finland; [4]Turku Center for Disease Modeling, Turku, Finland; [5]Department of Chemistry, University of Turku, Turku, Finland; [6]InFLAMES Research Flagship, University of Turku, Turku, Finland; [7]MediCity Research Laboratory, University of Turku, Turku, Finland; [8]Department of Gastroenterology, Turku University Hospital, Turku, Finland; [9]Turku PET Centre, Turku University Hospital, Turku, Finland.

[*]Equal contribution

Correspondence: Prof. Anne Roivainen, PhD, Turku PET Centre, Kiinamyllynkatu 4-8, FI-20520 Turku, Finland. Tel: +35823132862, E-mail: anne.roivainen@utu.fi

Assoc. Prof. Diana M. Toivola, PhD, E-mail: diana.toivola@abo.fi

Jukka Koffert, MD, PhD, E-mail: jukka.koffert@tyks.fi

First author: Achol A. Bhowmik, PhD student, Turku PET Centre, Kiinamyllynkatu 4–8, FI-20520 Turku, Finland. Tel: +358449832346, E-mail: achol.a.bhowmik@utu.fi

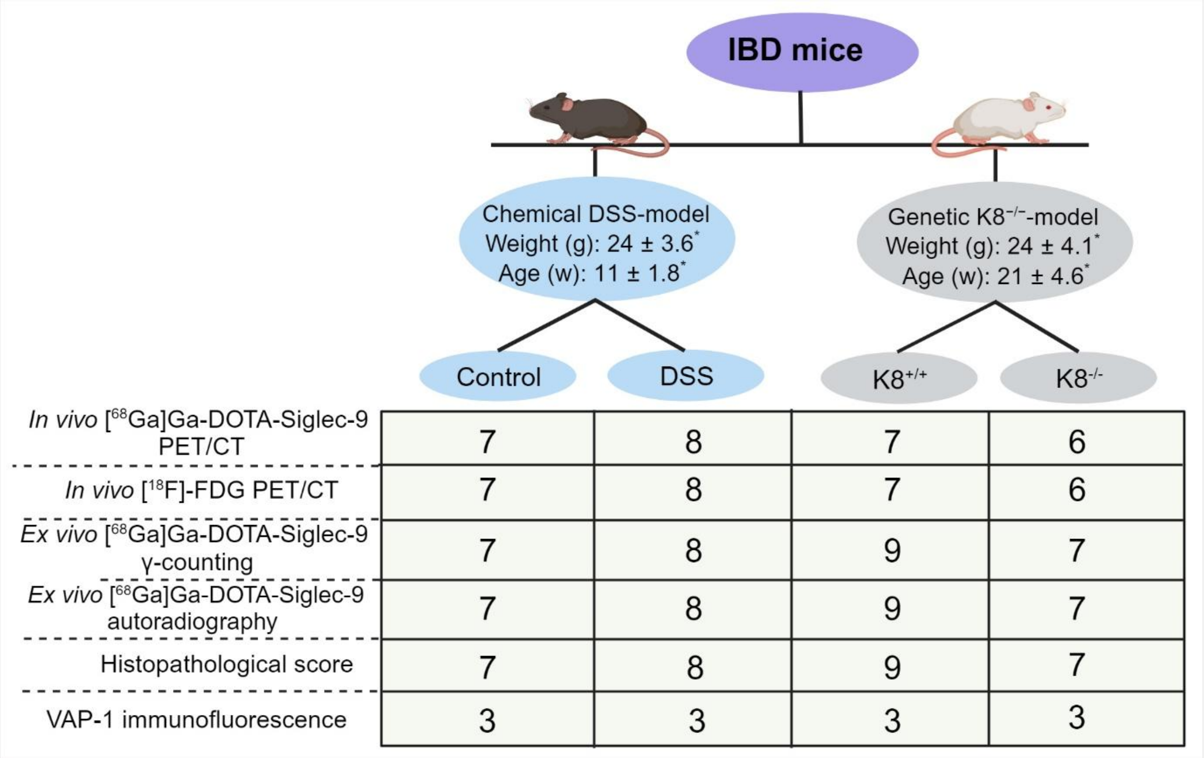

	Control	DSS	K8+/+	K8-/-
In vivo [^{68}Ga]Ga-DOTA-Siglec-9 PET/CT	7	8	7	6
In vivo [^{18}F]-FDG PET/CT	7	8	7	6
Ex vivo [^{68}Ga]Ga-DOTA-Siglec-9 γ-counting	7	8	9	7
Ex vivo [^{68}Ga]Ga-DOTA-Siglec-9 autoradiography	7	8	9	7
Histopathological score	7	8	9	7
VAP-1 immunofluorescence	3	3	3	3

Supplementary Fig. 1 Study flow chart and numbers of animals used. Male C57Bl/6Ncrl mice were used in the chemically-induced dextran sodium sulphate (DSS) model, and female FVB/n mice were used in the genetic keratin-8 ($K8^{-/-}$) model. *Values are expressed as mean ± SD.

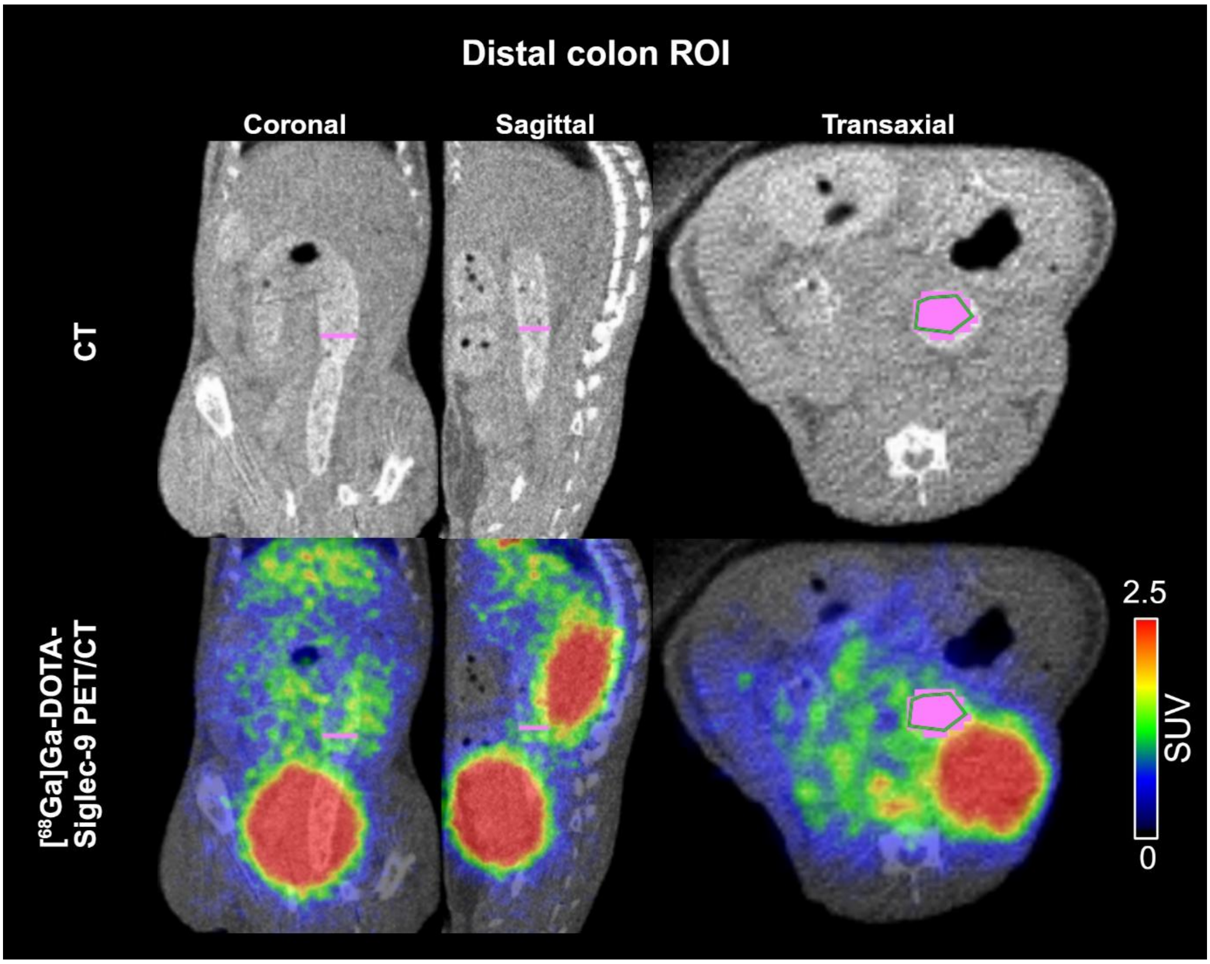

Supplementary Fig. 2 Representative contrast-enhanced CT images and fused [^{68}Ga]Ga-DOTA-Siglec-9 PET/CT images (time-weighted mean of PET frames from 0–60 min) of the same DSS male mouse as in Figure 3 showing region of interest (ROI) placement in the distal colon. ROI was manually defined on transaxial slice (pink region with green border) and the coronal and sagittal views (pink lines) were used to ensure the correct ROI placement. The size of ROI depended on cross-sectional area of the distal colon. The slice thickness was 0.4 mm. All ROIs were defined in three dimensions as shown in this example for distal colon ROI. CT images were utilized as anatomical reference. Distal colon ROIs were drawn avoiding spillover signal from the urinary bladder and kidneys. Mean SUV was calculated based on three consecutive transaxial slices.

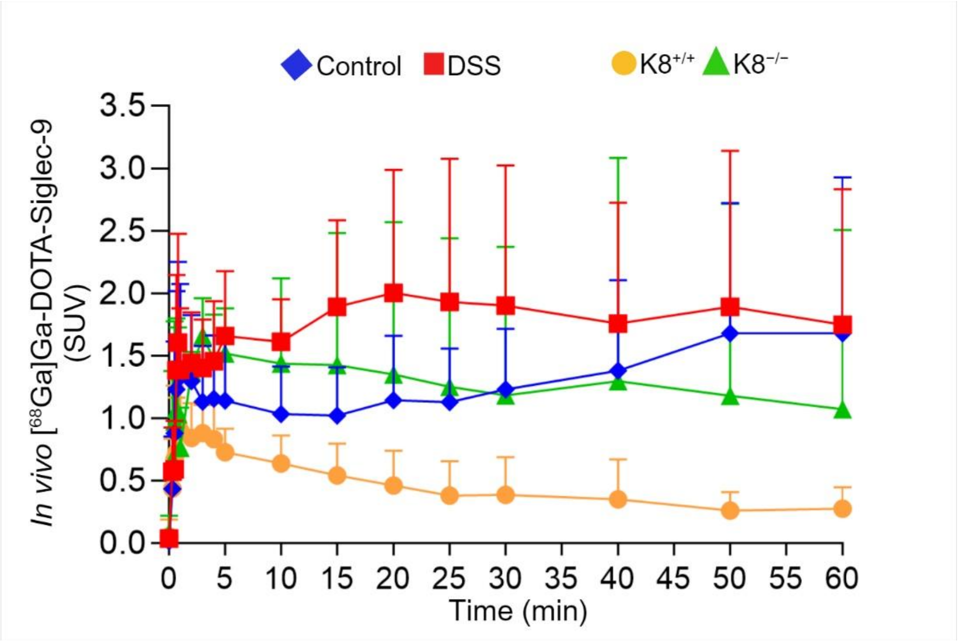

Supplementary Fig. 3 The decay-corrected time-activity curves of [^{68}Ga]Ga-DOTA-Siglec-9 uptake in the distal colon in different groups. Curves represent mean + SD (DSS *n* = 8, Control *n* = 7, K8$^{-/-}$ *n* = 6, K8$^{+/+}$ *n* = 7).
